# Reflections on qualitative research in global health: The practical complexities of crossing cultures, contexts, and languages

**DOI:** 10.7189/jogh.13.03012

**Published:** 2023-06-30

**Authors:** Olinda Santin, Hien Thi Ho, Chris Jenkins

**Affiliations:** 1 School of Nursing and Midwifery, Queen’s University Belfast, Belfast, Northern Ireland; 2 Faculty of Health and Medical Sciences, The University of Adelaide, Adelaide, South Australia, Australia; 3 Centre for Public Health, Queen’s University Belfast, Belfast, Northern Ireland

As researchers, we carry out our explorations while gripping tightly to the rulebook of methodologies. As an international and interdisciplinary team specializing in cancer care research, we have often however found ourselves without a “rulebook”, which prepared us for all eventualities and the fact that methods do not always translate between problems [1]. Qualitative research with people experiencing illness in different contexts, cultures, and languages is unpredictable, convoluted, and requires methodological flexibility. Adapting to obstacles quickly can be challenging, as research protocols by nature are pre-determined and often inflexible [2].

Here we share our experiences of bringing together researchers from Vietnam and Northern Ireland to qualitatively explore the support needs of families affected by cancer in Vietnam [3-5].

## DEVELOPING A QUALITATIVE METHODOLOGY – TIME AND CONSULTATION IS KEY

Principal investigators (PIs) often draft methodologies based on pre-conceived ideas of what may optimize data collection, which may negatively impact researchers working in countries in which they do not live. This became apparent early in our partnership, as researchers from high-income countries drafted protocols with little collaboration or consideration of the complexities of qualitative data collection in a low-income environment. Consequently, we made mistakes and developed impractical proposals that required significant amendments following grant awards.

Learning from our errors, we identified the need for our UK-based researchers to spend an extended period in Vietnam. We travelled from North to South, consulting with academics, patients, caregivers, charity groups, and healthcare professionals. We learned about local customs on how to show respect and facilitate openness, the optimal means to collect data, and how to promote research engagement for those lacking resources. These development activities provided the time and space to co-create interview schedules which were considerate of the complexities of language and phasing in the Vietnamese context.

We believe that pre-research visits and equitable collaboration are critical to the development of a qualitative methodology which promotes cultural competence, respect, and robust research, which can potentially impact health outcomes [6,7]. We were fortunate to have been granted institutional-led finances for this preparatory work; such opportunities are often limited, but we believe they are invaluable. In an environment in which financial support is often limited, the use of online platforms such as Zoom can be helpful in developing and sustaining partnerships. However, we believe in-person discussions are still key for early developmental work. Research funders and institutions should provide increased opportunities for “pre-study” work to promote quality qualitative research which is contextually and culturally appropriate.

## COLLECTING QUALITATIVE DATA – A TEAM APPROACH WORKS BEST

Once methods are drafted, we have found that the practicalities of collecting qualitative data require discussion and careful planning. In our early partnership, we often felt as though we were learning as we went through trial and error. We debated the merit of UK researchers/non-native speakers being present during interviews and focus groups. Given our earlier lessons, we were keen to ensure mutual understanding and equitable team roles [8]. Despite our apprehension and not speaking Vietnamese, we found it advantageous for our UK researchers to be present during interviews and focus groups. UK researchers assumed an observing/listening role, with Vietnamese researchers taking a lead role. Participants were comfortable with the presence of UK researchers and perceived their presence as an international acknowledgement of the importance of their experiences.

**Figure:**
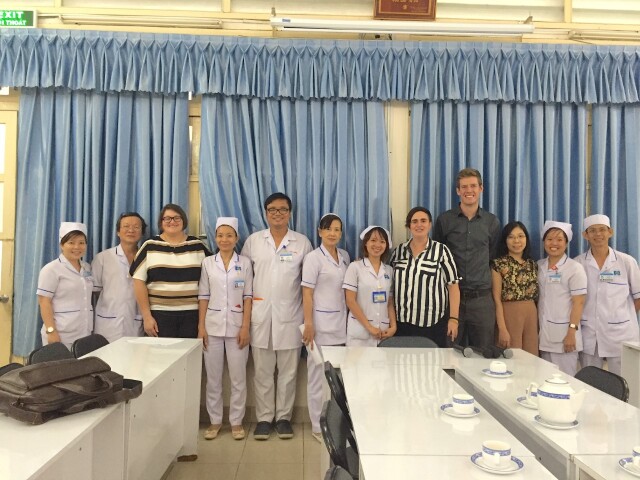
Photo: International research visits to develop qualitative research protocols. From Hien Thi Ho’s personal photo collection.

Data collection was conducted in Vietnamese by the PI and simultaneously translated by a second researcher to English-speaking researchers. UK researchers made detailed field notes and raised probes by writing notes or making comments to the translator/PI. Probing can be challenging to navigate due to cultural etiquette, while also avoiding asking leading questions [1]. We found that probes from the non-native-speaking researchers were helpful, as points were occasionally overlooked, since they were perceived as mundane by the Vietnamese-speaking team. Probing took some practice, however, as our early attempts often disrupted the interviewer’s flow. We found over time that managing probes allowed us to develop our discussions and increase our reflexivity. This process, however beneficial, relied heavily on our Vietnamese team’s fluency in English and was at times taxing on researchers who switched between the role of translator and researcher [9].

Despite being resource intensive, the presence of highly experienced qualitative researchers and PIs (both UK and Vietnam) was key to ensuring the depth of data collection. These practices improved the quality of our studies, while simultaneously building research skills and capacity for early career researchers.

Due to travel and institutional commitments, we collected our data in a two-week period, resulting in researcher fatigue, which is far from ideal in a qualitative context. If possible, extended time and resources should be allowed to facilitate the presence of all team members during data collection.

## DATA ANALYSIS – EXTRA TIME AND EXTRA STEPS ARE NEEDED

Interpretation of data across languages, cultures, and contexts is difficult, and we often faced misunderstandings. Factoring in additional time and steps was key to overcoming these challenges [10]. Concurrent analysis, particularly for non-native researchers, is necessary for reducing potential inaccuracies or misrepresentations of views. Following each focus group or interview, non-native researchers provided detailed notes and a written and verbal overview of the key themes or subthemes to the larger team. This process of inter-rater reliability and feedback provided additional context and meaning and continued until data collection was finished. Conducting this step within the country strengthened data quality and trustworthiness and allowed us to identify and correct any challenges or misconceptions. This process was also supported by a detailed translation of transcripts into English.

We proceeded through all the usual steps of qualitative analysis, including, *verbatim* transcription, member checking, back translation to English, coding, and development of themes and subthemes. We found that analysis is more robust when conducted face to face, with the whole research team reading, coding, and theming data in an iterative discussion. Our Vietnamese team spent time in the UK developing the analysis in order to improve it.

## PREPARING MANUSCRIPTS – KEEP TALKING

We identified many misinterpretations or lack of depth in analysis during the writing process. As our partnership developed, we learned the important step of verbalizing our interpretation to each other. Each member, in turn, discussed their data interpretation, with the listener paraphrasing back to ensure understanding of perspective. Verbalizing data interpretation opens discussions, adds depth to interpretation, and aids consensus. Results are often written as a team discussing the interpretation line-by-line until consensus is reached and the manuscript finalized. This process requires experience and leadership and is not without practical implications; it depends on financial and time resources, team motivation, and requires long periods of time spent overseas. We found that English-speaking researchers should provide time and space for supporting the writing process, especially redrafting paragraphs and sentences to ensure alignment with research findings, thus increasing the trustworthiness of data.

## CONCLUSIONS

Qualitative global health research is essential for understanding and responding to illness and disease within context. Qualitative research methods are not culturally neutral, and the joining of disciplines, languages, and contexts can present many challenges. Researchers need to discuss the practicalities of doing qualitative research across contexts and languages, as extra consideration and steps are necessary to ensure major pitfalls.
